# Dr. John Bibby

**Published:** 1911-04-15

**Authors:** 


### Obituary.

The death is announced on April 8, at Holland Road, London, of Dr. John Bibby, aged fifty-six years. Dr. Bibby, who has been much abroad of recent years, took his M.R.C.S. in 1878, and his L.R.C.P. (Ireland) in 1895. He studied at Liverpool and St. Thomas' Hospital, and held the D.P.H. of Cambridge. He had been house-surgeon at Liverpool Royal Infirmary.

